# Dietary supplementation with a specific melon concentrate reverses vascular dysfunction induced by cafeteria diet

**DOI:** 10.3402/fnr.v60.32729

**Published:** 2016-11-07

**Authors:** Julie Carillon, Bernard Jover, Jean-Paul Cristol, Jean-Max Rouanet, Sylvain Richard, Anne Virsolvy

**Affiliations:** 1 Nutrition & Metabolism, UMR 204 NutriPass, University of Montpellier, Montpellier, France; 2 Bionov Research, Montpellier, France; 3 EA7288, University of Montpellier, Montpellier, France; 4 PhyMedExp, INSERM U1046, UMR CNRS 9214, University of Montpellier, Montpellier, France

**Keywords:** vascular function, oxidative stress, antioxidant, superoxide dismutase, obesity

## Abstract

**Background:**

Obesity-related metabolic syndrome is associated with high incidence of cardiovascular diseases partially consecutive to vascular dysfunction. Therapeutic strategies consisting of multidisciplinary interventions include nutritional approaches. Benefits of supplementation with a specific melon concentrate, enriched in superoxide dismutase (SOD), have previously been shown on the development of insulin resistance and inflammation in a nutritional hamster model of obesity.

**Objective:**

We further investigated arterial function in this animal model of metabolic syndrome and studied the effect of melon concentrate supplementation on arterial contractile activity.

**Design and results:**

The study was performed on a hamster model of diet-induced obesity. After a 15-week period of cafeteria diet, animals were supplemented during 4 weeks with a specific melon concentrate (*Cucumis melo* L.) Contractile responses of isolated aorta to various agonists and antagonists were studied *ex vivo*. Cafeteria diet induced vascular contractile dysfunction associated with morphological remodeling. Melon concentrate supplementation partially corrected these dysfunctions; reduced morphological alterations; and improved contractile function, especially by increasing nitric oxide bioavailability and expression of endogenous SOD.

**Conclusions:**

Supplementation with the specific melon concentrate improves vascular dysfunction associated with obesity. This beneficial effect may be accounted for by induction of endogenous antioxidant defense. Such an approach in line with nutritional interventions could be a useful strategy to manage metabolic syndrome–induced cardiovascular trouble.

Human obesity is not only consecutive to excessive saturated fat intake, but also results from a complex combination of multiple nutritional and lifestyle-related factors directly linked to the excessive consumption of industrial era foods (1). The modern Western lifestyle, merging stress, low-quality food (rich in fat and energy, but poor in micronutrients), and the disruption of chronobiological function/rhythms, contributes to the increase of metabolic syndrome incidence (2). Diets consisting of palatable industrially processed foods (named cafeteria diets) are suitable for modeling in animals the metabolic disorders of human obesity. Indeed, these cafeteria models lead to a phenotype of exaggerated obesity and related disorders similar to those responsible of metabolic syndrome (3).

The metabolic syndrome combines multifactorial health issues, such as glucose intolerance, central obesity, dyslipidemia, and insulin resistance (4), and it is frequently associated with hypertension. These conditions increase the risk of development of several pathologies, especially cardiovascular diseases and type 2 diabetes (5–7). Vascular disorders are central, involving morphological and functional remodeling of the arterial vasculature. Hemodynamic parameters are altered that imposes stress in the heart and other organs (8). Major changes are arterial stiffening (9), arterial wall thickening, and endothelial dysfunction (10). In the presence of physiological and pathological stimuli, the components of the arterial wall reorganize to maintain the integrity of the vessel wall, and these changes could increase the potential for vascular dysfunction (8).

Oxidative stress, associated with many components of metabolic syndrome, plays an important role in the pathogenesis of vascular dysfunction (11, 12). Oxidative stress results from an imbalance between production and inactivation of reactive oxygen species (ROS), which contributes to cellular dysfunction. An elevation in ROS production is associated with endothelial dysfunction, and vascular ROS may play a role in the development of obesity and metabolic syndrome (13).

Antioxidant dietary supplementations have beneficial effects on vascular dysfunction (14, 15). Supporting the efficiency of such nutritional approaches is the increasing emphasis on the positive effects of micronutrients found in natural products for controlling the pathogenesis of chronic disease such as metabolic syndrome (16).

Since 2000, a proprietary melon juice concentrate containing a high level of superoxide dismutase (SOD) has been developed. Its use as a dietary supplement has been a new subject of interest, and its antioxidant and anti-inflammatory properties have been demonstrated (17–21). Recently, it was reported that supplementation with this melon concentrate increases endogenous antioxidant defenses and thus reduces oxidative stress, insulin resistance, and corrected adipose tissue alterations in a hamster model of metabolic syndrome (18). Beneficial cardiac effects, correlated with induction of endogenous antioxidant defenses, were also observed in a rat model of cardiac hypertrophy (22, 23). Oxidative stress has been associated with the onset of cardiovascular complications in subjects with metabolic syndrome and especially with vascular dysfunction (24). In that context, information regarding the potential effects of this melon concentrate on vascular alterations was lacking.

Currently, the therapeutic strategy to specifically treat metabolic syndrome consists of multidisciplinary interventions including nutritional approaches. As many issues associated with obesity are mediated by oxidative stress, the use of a dietary antioxidant derived from a natural food source to antagonize such damage offers great potential.

In this work, we hypothesized that this specific melon concentrate can reverse vascular morphological and functional alterations associated with obesity through antioxidant properties. We investigated the effect of a 1-month oral supplementation with the melon concentrate on vascular function in a Golden Syrian hamster model of diet-induced metabolic syndrome. Supplementation was given to animals fed for 3 months with a diet consisting of high-fat, high-sugar, and high-salt supermarket products (Western diet). Endpoint direct arterial pressure was measured. Measurements of vascular reactivity and morphological analysis of thoracic aorta were performed to evaluate obesity-induced alterations. Oxidative status was also determined to understand the mechanism.

## Materials and methods

### Preparation and characterization of the melon concentrate

SODB (Bionov, Avignon, France) is a dried melon juice concentrate that is particularly rich in SOD, as a result of a patented process.

Approximately 625 kg of a specific proprietary and no–genetically modified organism melon variety *Cucumis melo* L. (equivalent to 15 kg of dried melon pulp) is needed to produce 1 kg of this dried melon juice concentrate. In brief, the melon pulp is separated from skin and seeds and crushed before centrifugation. Then, the melon juice undergoes filtration and concentration steps. Finally, the obtained melon juice concentrate is freeze-dried. For nutraceutical applications, this freeze-dried melon juice concentrate is coated with palm oil by spray drying method to preserve SOD activity from the digestive enzymes secreted above the small intestine. Detailed information about the antioxidant content of this dried melon juice concentrate has been published in a previous study (25).

### Experimental design

The present animal experiments complied with European and French laws conformed to the *Guide for the Care and Use of Laboratory Animals, 8th ed*. (2011) published by the National Institutes of Health (National Academies Press, Washington, DC) and were approved by the Committee for Animal Care at the University of Montpellier (France) (permission no. C 34 249).

Eighteen 3-week-old male Golden Syrian hamsters (Janvier, Le Genest-St-Isle, France) were used. They were housed at 23±1°C and subjected to a 12-h light/dark cycle, with free access to both food and water. After an 18-day adaptation period, the hamsters (75–80 g) were randomly divided into three groups. Two groups of hamsters (*n=*6 per group) were assigned for 19 weeks to a cafeteria diet consisting of nine types of palatable industrially processed foods designed for human consumption (e.g. cake, potato crisps, sweets, cheese) and selected for their high-energy, -fat, -sugar and/or -salt content. The cafeteria items were weighed before being presented to the hamsters, and they were provided in excess, thereby inducing obesity. Detailed information about the nutritional value and ingredients of all foodstuffs used in this diet are presented in our previous study (17). After 15 weeks, one of the two groups of obese animals was orally supplemented with the melon concentrate (10 U SOD equivalent/day, corresponding to 10 mg/day, mixed with food) for the last 4 weeks (OB-treated group), whereas the other group was maintained on the cafeteria diet (OB group). The third group of hamsters (*n=*6) received a standard pelleted diet (EF Hamster Control E21000-04, SSNIFF, Soest, Germany) and served as control (standard [STD] group). Food intake and body weight were recorded daily.

### Direct arterial pressure measurement

At the end of the experimental protocol and the day of sacrifice, systolic arterial pressure was measured in the carotid artery. Hamsters were anesthetized (ketamine and xylazine, 75 and 25 mg/kg, respectively), and a polyethylene catheter (PE-50) was inserted into the right carotid artery for arterial pressure recordings.

### Vascular reactivity

The thoracic aorta was used to study *ex vivo* the responses to agonists and antagonists of arterial contraction. Immediately after removal, arterial tissue was immersed in phosphate saline solution (PSS), pH 7.4, containing (in mM) 140 NaCl, 5 KCl, 1 MgCl_2_, 0.5 KH_2_PO_4_, 0.5 Na_2_HPO_4_, 2.5 CaCl_2_, 10 HEPES, and 10 glucose. Aortic tissue was cleaned of fat and connective tissue and cut into 2–3-mm-wide rings. Aortic rings were mounted in standard organ bath chambers (EMKA Technologies, Paris, France) maintained at 37°C and continuously bubbled with O_2_. Then, changes in isometric tension were recorded as described previously (26). Each arterial segment was subjected to a 60-min equilibration period at the predetermined optimal basal tension of 1 g. The contractile function of each segment was assessed with 1 µM phenylephrine (PE), and the presence of endothelium was confirmed by the vasorelaxation induced by application of acetylcholine (Ach, 1 µM). After several washouts and a 20–30-min period of stabilization, dose responses were performed by cumulative increases in the concentration of the agonist PE (0.01–100 µM range) or the depolarizing agent KCl (1–80 mM). Endothelial function was assessed by studying the relaxing effects of cumulative increases of Ach between 1 nM and 10 µM on arteries contracted with a submaximally active concentration of PE (10 µM). The effects of the nitric oxide (NO)-synthase inhibitor *N* ^*ω*^-nitro-l-arginine methyl ester (L-NAME, 10 µM) and the ROS scavenger tempol (10 µM) were evaluated on the relaxing effect of Ach. Inhibitors were added for a 15-min period of incubation before PE addition. Endothelium-independent relaxations to sodium nitroprusside (SNP, 1 nM–200 µM) were studied in endothelium-denuded rings previously contracted with PE (10 µM). Each protocol was performed in triplicate for each aorta. All chemical compounds were purchased from Sigma-Aldrich (Saint Quentin Fallavier, France).

### Aortic morphology

Thickness and internal diameter determinations were performed according to two methods. First, aortic rings were immersed in PSS, and optical images of the aortic cross-section were recorded to allow determination of basal internal diameter and thickness. Second, light microscope images were taken after hematoxylin-eosin staining realized on paraffin-embedded tissue sections. The mean internal aortic diameter and media thickness were determined from three measures realized on at least four arterial segments per animal by using ImageJ (National Institutes of Health, Bethesda, MD). Media cross-sectional area was calculated from these measures. Both methods gave similar results, and data represent means of all values obtained.

### Determination of aortic SOD expression by real-time PCR

Total RNAs were extracted from frozen aorta using Trizol^®^ (Sigma-Aldrich) according to manufacturer's instructions. DNase-treated (DNase I, Invitrogen, Saint Aubin, France) total RNA (1–2 µg) was transcribed into cDNA by using SuperScript II reverse transcriptase (Invitrogen) and random primer oligonucleotides (Invitrogen). Gene-specific primers for SOD3 and the housekeeping gene glyceraldehyde 3-phosphate dehydrogenase (GAPDH) were designed using the Universal Probe Library Assay Design center (Roche, Boulogne-Billancourt, France) and are as follows: SOD3-forward, 5′-tgtatgcaatctgccaggtg-3′; SOD3-reverse, 5′-tgacagctgcttgaagagga-3′; GAPDH-forward, 5′-tggctacagcaacagagtgg-3′, and GAPDH-reverse, 5′-ggggttattggacagggact-3′. Real-time quantitative polymerase chain reaction (PCR) was performed in a LightCycler System (Roche) in combination with the Absolute QPCR SYBR Green Capillary mix (Thermo Fisher Scientific, Illkirch, France). After a hot start (5 min at 95°C), the parameters for amplification were as follows: 10 sec at 95°C, 10 sec at 58°C, and 15 sec at 72°C for 50 cycles. Primers (Table 1) selected were of equal efficiency (E_ff_=1.9) across the range of template concentrations (1–10 ng of cDNA). Expression levels normalized with GAPDH were calculated relative to the less abundant isoform (Na_v_1.6) by using the E_ff_^−ΔΔCt^ method.

**Table 1:** Body weight and blood pressure of animals for various diets

	STD	OB	OB treated
Body weight at 15 weeks (g)	111±2	195±3*	195±2*
Body weight at 19 weeks (g)	114±2	200±2*	188±2*§
Systolic arterial pressure (mmHg**)**	136±15	135±8	134±13

STD=standard diet, OB=cafeteria diet, OB treated=cafeteria diet plus melon concentrate supplementation. Values are presented as mean±SEM.

* *p<*0.05, OB and OB-treated vs. STD animals

§ *p<*0.05, effect of the melon concentrate.

### Statistical analyses

Values are presented as mean±standard error of the mean (SEM). Statistical analysis of the data was carried out using Prism^®^ software (GraphPad, La Jolla, CA) by one-way analysis of variance (ANOVA) followed by a Mann–Whitney protected least significant difference test. For dose–response curves, data were analyzed by two-way ANOVA followed by Bonferroni post-test. *p* values<0.05 were considered to be significant.

## Results

### Melon concentrate supplementation reduced body weight gain

Cafeteria diet induced a significant increase in body weight (OB group) compared with the standard diet (STD; Table 1). The melon concentrate supplementation slightly decreased body weight (5% lower than in the untreated OB group), although food intake was not affected. As shown in Table 1, systolic arterial pressure was similar in all groups.

### Melon concentrate supplementation improved morphological alterations of aorta

**Fig. 1:**
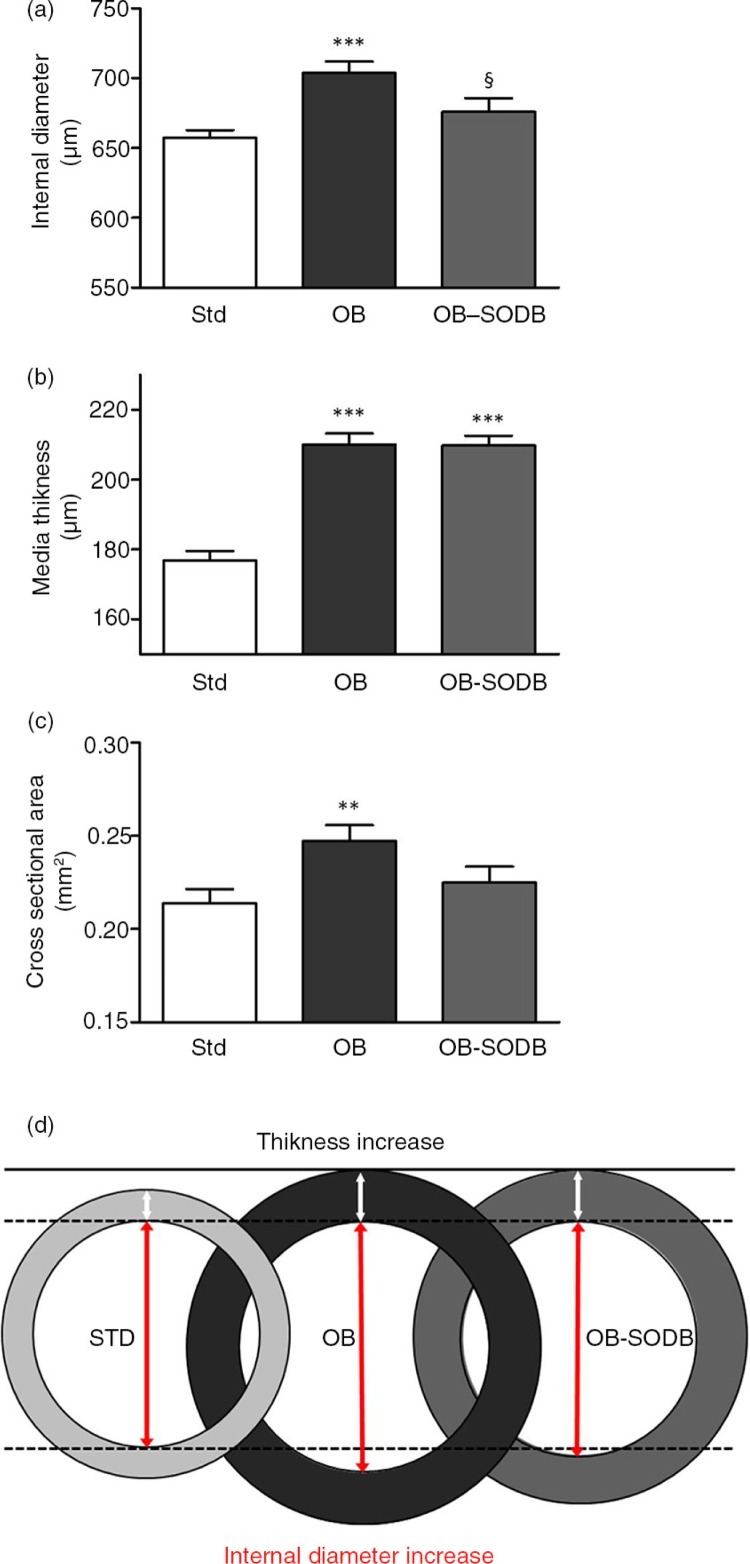
Morphological changes in aorta isolated from STD, OB, and OB-treated animals. (a) Intraluminal diameters. (b) Mean thickness of aortic media. (c) Cross-sectional area calculated from values obtained for internal diameter and media thickness for each arterial segment. (d) Schematic representation of aorta cross-section illustrates changes for each group: STD, OB, and OB-treated animals. Data are presented as mean±SEM (*n=*6 hamsters in each group). At least four segments per animal were analyzed. **p<0.01, ***p<0.001, OB and OB treated vs. STD animals; §§*p<*0.01, effect of the melon concentrate.

In animals subjected to cafeteria diet (OB group), we observed increases in both internal diameter and media thickness of aorta (Fig. 1), corresponding to 7±1% (*p*=0.0007) and 19±2% (*p*<0.0001), respectively, compared with the same parameters measured in the reference STD group. Consequently, the cross-sectional area was 16±4% higher in the OB group than in the STD group (*p=*0.0318). The melon concentrate supplementation tended to reverse the increase in internal diameter (Fig. 1a; *p=*0.0581) induced by the cafeteria diet in the OB-treated group; no difference was observed with STD group (*p=*0.2564). In contrast, the melon concentrate supplementation had no effect on the media thickness in the OB group (Fig. 1b). As a result, the cross-sectional area in the OB-treated group was intermediate between that of the OB and that of the STD group (Fig. 1c). Thus, arterial remodeling induced by cafeteria diet reflected outward hypertrophic remodeling, partially reversed by the melon concentrate supplementation (Fig. 1d).

### Melon concentrate supplementation corrected alterations of vascular contractility

**Fig. 2:**
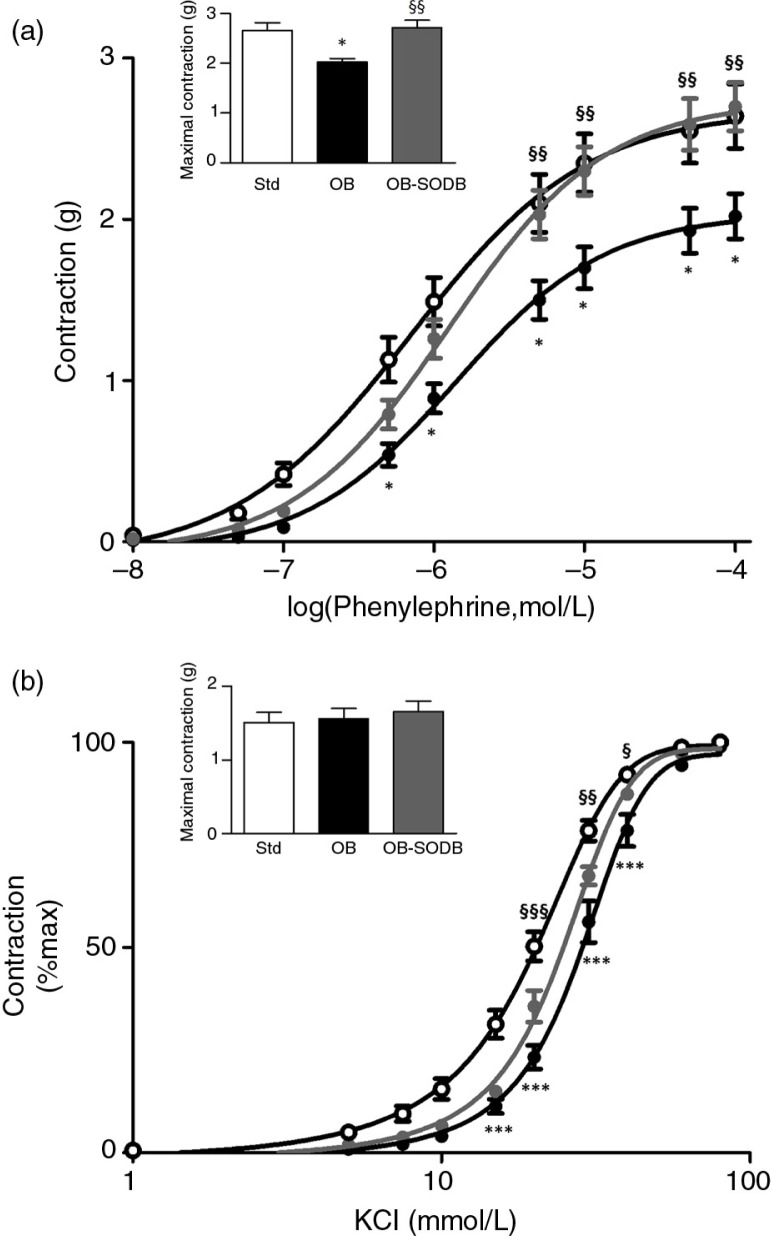
Contractile responses of hamster aorta. Graphs represent cumulative response curves (a) to PE (0.01–100 µM) and (b) to KCl (1–80 mM) for aorta isolated from STD, OB, and OB-treated animals. Insets represent the maximal contraction induced by a maximally active concentration of PE (a) and KCl (b). Values are presented as mean±SEM (*n=*6). *p<0.05, ***p<0.001, OB and OB-treated vs. STD animals; §*p<*0.05, §§*p<*0.01, §§§*p<*0.001 vs. effect of the melon concentrate.

PE and KCl induced robust dose-dependent vasoconstriction of hamster aorta (Fig. 2). PE promoted contractile response weaker in the OB group than in the STD group, irrespective of the concentration, and the EC_50_ value was increased (0.82±0.16 µM in STD vs. 1.5±0.16 µM in OB; *p=*0.0079) (Fig. 2a). Maximal PE-induced contraction was decreased by 24±3% (*p=*0.0102). The melon concentrate supplementation fully corrected the loss of aortic contraction (*p=*0.004, OB treated vs. OB) with a partial recovery of half maximal effective concentration (EC_50_) value (1.21±0.26 µM; *p=*0.0506). The maximal responses to PE were identical in the OB-treated and STD groups (Fig. 2a; *p=*0.9661). Cafeteria diet also induced modifications in the responses to KCl (Fig. 2b). We observed a rightward shift of the dose–response curve for OB group. With no difference in the maximal KCl-induced contraction. The EC_50_ value was increased reflecting lower sensitivity to KCl depolarization (29.4±1.7 for OB vs. 20.4 ±1.1 mM for STD; *p<*0.0001). The melon concentrate supplementation corrected this shift (EC_50_=24.5±0.8 mM; *p=*0.0495, OB treated vs. OB). No significant difference in KCl sensitivity was detected compared to STD (*p=*0.0985).

### Melon concentrate supplementation corrected alterations of vasorelaxation

**Fig. 3:**
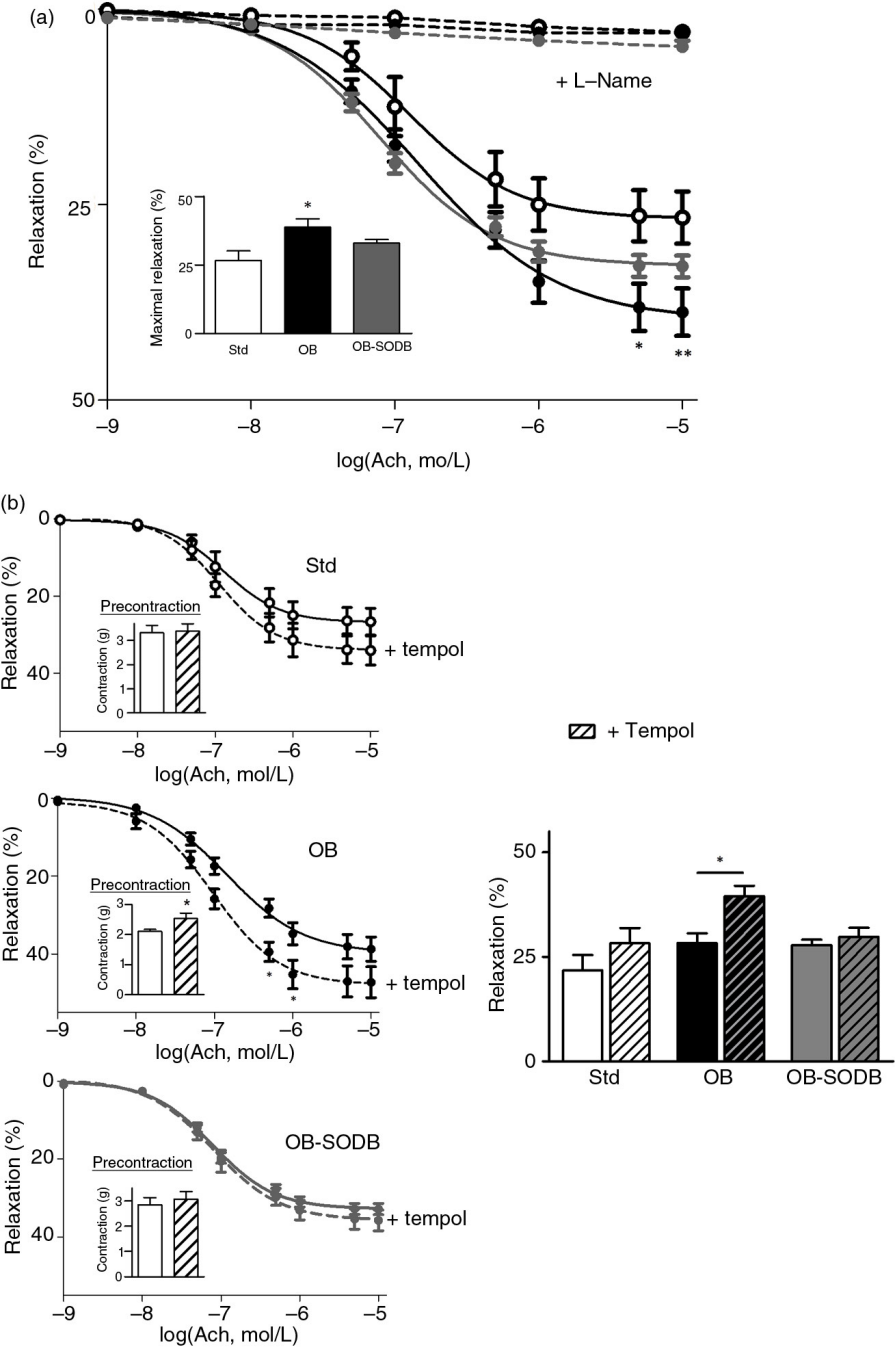
Ach-mediated relaxation. Graphs represent cumulative dose–response curves to Ach, in the presence of endothelium, in aortic rings previously contracted with a submaximally active concentration of PE (10 µM). Data are expressed as percentage of relaxation relatively to the contraction induced by PE. (a) Relaxing effects of cumulative concentrations of Ach were studied in the absence (plain lines) and in the presence (dotted lines) of l-NAME (10 µM). The inset shows the maximal relaxing effect of Ach (10 µM) for each group (STD, OB, and OB treated). (b) Effect of PE and Ach was evaluated in the presence of tempol (10 µM). Dose–response curves for Ach are shown for each group; the insets represent the previous contractions obtained for PE before Ach addition and for both conditions absence and presence of tempol. The bar graph summarizes the relaxing effects of 1 µM Ach in the absence and in the presence of tempol for STD, OB, and OB-treated groups. Values are presented as mean±SEM (*n=*6). *p<0.05, **p<0.01, OB and OB-treated vs. STD animals.

**Fig. 4:**
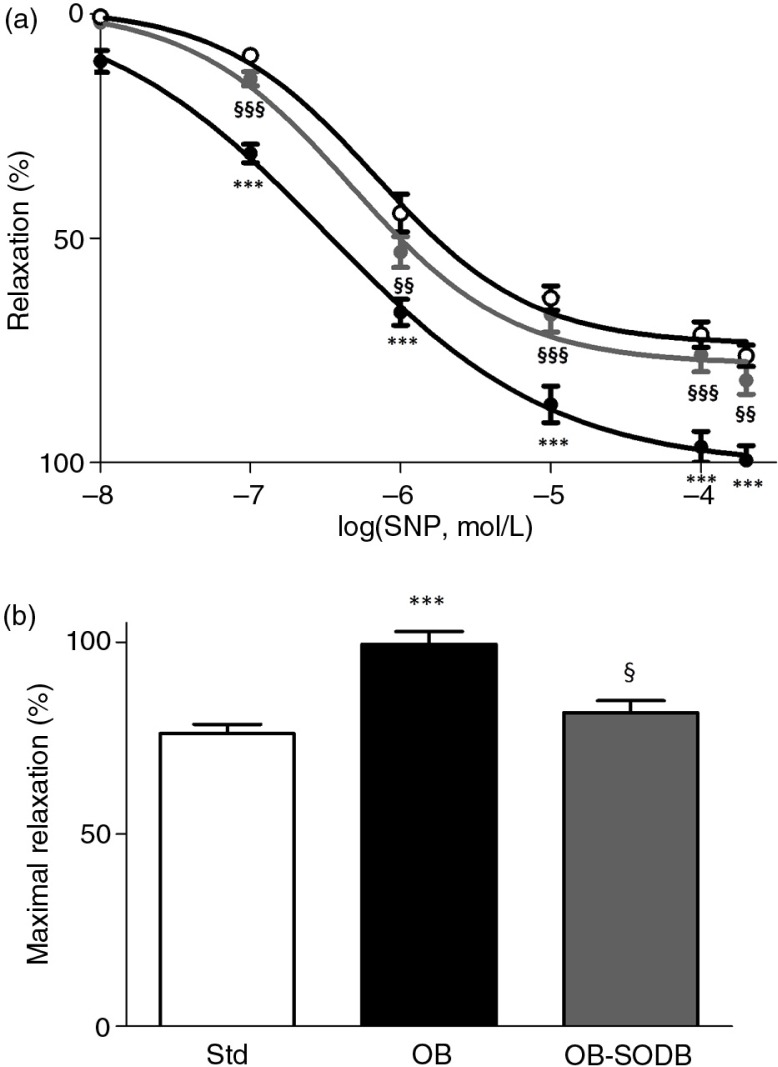
SNP-induced endothelium-independent vasorelaxation. (a) Cumulative dose–response curves to SNP (0.01–200 µM) of aortic rings endothelium denuded and previously contracted with PE (10 µM) in STD, OB, and OB-treated groups. Data are expressed as percentage of relaxation relatively to the contraction induced by PE. (b) Bar graph represents the maximal relaxation obtained for each group. Values are presented as mean±SEM. ***p<0.001, OB and OB-treated vs. STD animals; §*p<*0.05, §§*p<*0.01, §§§*p<*0.001 vs. effect of the melon concentrate.

Ach and SNP response were investigated to evaluate endothelium-dependent and -independent vasorelaxation, respectively. Both Ach and SNP induced vasorelaxation of hamster aorta pre-contracted with a submaximal dose of PE (Figs. 3 and 4). Ach-induced relaxation was higher in the OB compared to the STD group (*p=*0.0121). The increase in maximal Ach response corresponded to 39±2% of the response in the STD group (Fig. 3a). The melon concentrate supplementation tended to normalize the Ach-mediated relaxant properties. In the OB-treated group, Ach-induced relaxation was intermediate, with a trend to be different than in the OB group (*p=*0.0593) and to be normalized as the STD (*p=*0.0691). The presence of L-NAME suppressed the relaxant effect of Ach similarly whatever the diet.

The same experiments performed in the presence of tempol, a ROS scavenger, showed that Ach-induced relaxation was maintained in the STD and the OB-treated group, but it was potentiated in OB group (Fig. 3b) compared to Ach-induced relaxation in absence of tempol. Indeed, in the OB group, tempol amplified the maximal response to Ach, with a 40% increase (in absence vs. in presence of tempol; *p=*0.0209). This potentiation was absent in the STD group (*p=*0.3198) and was suppressed by the melon concentrate supplementation (OB treated, *p>*0.9999). In the presence of tempol, PE response was also potentiated in OB group, whereas no potentiation was observed in STD as in OB treated (Fig. 3b, insets).

SNP dose dependently relaxed arteries previously contracted with a submaximally active concentration of PE (Fig. 4a). Cafeteria diet induced a potentiation of that relaxation (Fig. 4b). In the OB group, the maximal vasorelaxant capacity of aorta to SNP was increased by 30% compared to the STD group (*p=*0.0004). The melon concentrate supplementation suppressed the potentiation of the SNP response (*p=*0.0125) and in the OB-treated group, relaxation of aorta was identical to that of the STD group (*p=*0.3818).

### Melon concentrate supplementation modulated aortic oxidative status

**Fig. 5:**
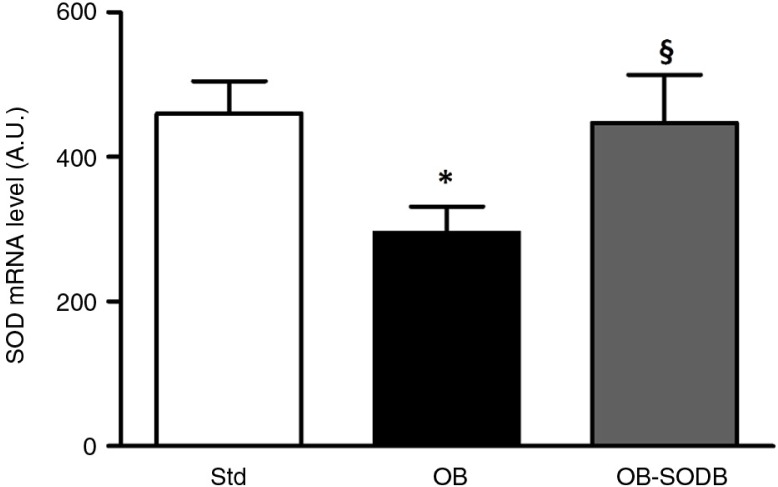
Expression of aortic SOD. SOD3 mRNA levels were quantified by real-time PCR in aorta. GAPDH was used to normalize total RNA amount. Relative levels were presented in arbitrary units as mean±SEM of two determinations in all animals in each group (*n=*6). *p<0.05, OB and OB-treated vs. STD animals; §*p<*0.05 vs. effect of the melon concentrate.

Expression of antioxidant enzymes and especially SOD could be modified by cafeteria diet. In our model, we observed that SOD mRNA level was significantly lower in the OB group (Fig. 5). It was decreased by 34% compared to the STD group (*p=*0.0318). The melon concentrate supplementation corrected this parameter. In OB-treated group, the endogenous SOD level, which was higher than that of the OB group (*p=*0.0439), did not differ from that of the STD group.

## Discussion

The beneficial effects of dietary supplementation with the melon concentrate reported in various animal and human models are currently presented as consecutive to a reduction of oxidative stress in relation with an increase in the endogenous antioxidant defenses, especially SOD (17, 18, 22). The present study extends this concept to the vascular level. In our hamster model, cafeteria diet induced vascular alterations mainly characterized by tissue remodeling, contractile dysfunction, and oxidative stress. A dietary supplementation with the particular melon concentrate corrected these disorders.

We have previously reported that, in the same animal model, cafeteria diet induces obesity and various related disorders such as dyslipidemia, insulin resistance, oxidative stress, or adipose tissue fibrosis that mimic metabolic syndrome disorders in humans (17, 18). Presently, we observed that the cafeteria diet induced a morphological remodeling characterized by increases in both intraluminal diameter and media thickness of large arteries. These modifications reflect outward hypertrophic remodeling as shown in both human and animal models (9, 27). It is seen as an adaptive mechanism associated with metabolic syndrome and consecutive to flow-mediated shear stress (28, 29). At first, these adaptations are compensatory and aim to ensure adequate tissue perfusion in relation to higher metabolic requirement. However, in later stages, they become detrimental and contribute to accelerated risk for cardiovascular disorders (8). Increased blood flow and hypertension, usually associated with obesity, are correlated to arterial stiffness (8, 30). At high blood pressure, vascular-wall stress is prominent and in arteries, less compliant collagen fibers predominate. Abnormalities of arterial function described in humans include both endothelium-dependent and -independent vasodilatation (31). In animal models, vascular reactivity abnormalities mainly consisted of increased contractility and reduced vasodilation (32). Here, in our hamster model of diet-induced obesity, arterial dysfunction was characterized by decreased contractile and increased vasodilatory responses. Such alterations and absence of arterial pressure elevation after high-fat diet have been otherwise reported in another kind of muscular arteries, coronary arteries (33–36). These discrepancies remain unexplained, but they could be due to differences in the disease state studied, or related to diet and different models. Nevertheless, the vascular dysfunction observed in OB group is consistent with the absence of hypertension and revealed an adaptive response resulting in an enhancement of vasorelaxation and a decrease in contractility to maintain adequate tissue perfusion. As a major result of this work, we showed that the melon concentrate supplementation partially reversed the vascular remodeling and improved arterial function. The improvement of arterial function was illustrated by the contractile and vasorelaxant responses in OB-treated group compared to the OB group.

Vasomotor responses are the result of smooth muscle activity regulated by the endothelium. Impaired vascular function can involve endothelial and smooth muscle dysfunction. A high-fat diet is usually related to endothelium dysfunction and reduced NO bioactivity (37). In our model, we observed that both Ach- and SNP-induced relaxations were enhanced in the OB group. This suggests that increased sensitivity to NO, rather than endothelium dysfunction, occurred. Indeed, SNP-induced relaxation is NO dependent, but endothelium independent, whereas Ach induced an NO- and endothelium-dependent relaxation. Enhanced vasodilator capacity of vessels in response to NO and SNP has been previously reported in some models of obesity (33, 36). This phenomenon, thought to be an adaptive mechanism providing adequate tissue perfusion, has been shown to involve increased NO sensitivity that is probably associated with impaired NO bioavailability (34, 38). We observed that the ROS scavenger tempol potentiated Ach-induced relaxation and PE-induced contraction in the OB group. Thus, in the presence of tempol, PE-induced contraction otherwise decreased in the OB group tended to be normalized and identical to STD animals. Tempol is known to prevent NO degradation and thus increases NO bioavailability (39) and mimics the effect of endogenous cellular defense. The effect of tempol on vasomotor responses in the OB group unmasked an increased NO degradation that was probably related to high level of cellular ROS and oxidative stress, as observed previously (18). One major cellular defense against oxidative stress is a group of oxidoreductases known as SODs. SODs catalyze the dismutation of O_2_^•−^ into H_2_O_2_ and oxygen and thus prevent the inactivation of NO. In blood vessel walls and particularly in the arteries, extracellular SOD (or SOD3) is the main antioxidant defense enzyme (39). Interestingly, the SOD3 mRNA level was decreased in the OB group compared to the STD group. Thus, cafeteria diet triggered vascular disorders related to increased oxidative stress and decreased antioxidant defenses.

The potentiating effect of tempol on Ach-induced relaxation was not observed in the OB-treated group like in the STD group, consistently with a low level of oxidative stress. Thus, the melon concentrate supplementation seemed to correct the decrease in antioxidant defense. This was corroborated by an SOD3 mRNA level that was higher in the OB-treated group than in the OB group and not different from that of the STD group. Vascular SOD3 is localized in a high concentration between the endothelium and the smooth muscle. This localization, at the extracellular matrix level and endothelial cell surfaces, confers a critical role for SOD3 in preventing oxidation of NO released by the endothelium, which is of importance in the regulation of NO bioavailability (40). The beneficial effect of the melon concentrate supplementation on vascular function could be accounted for by an increase in NO bioavailability through SOD3 up-regulation. Consequently, this is associated with a reduction in ROS production that is probably related to a reduced inflammatory status and is consistent with our previous observations (17, 22).

We observed in our model that the melon concentrate supplementation triggers endogenous SOD3 expression. This is in line with previous studies reporting increased levels of antioxidant enzymes in various animal models after melon concentrate supplementation (17, 22, 41). The precise mechanism of the induction remains to be clarified and warrants further investigation. Although the melon concentrate contains a high level of SOD, a direct effect of the enzyme is not considered because its high molecular weight excludes intestinal absorption. Currently, this particular melon concentrate is thought to trigger a cascade of events that initiates the induction of antioxidant defense in various tissues from the intestine through a local stimulation of the immune system (42). The natural product contains active ingredients that will activate antioxidant defense of the host, with a systemic effect at multiple organ levels.

Our results suggest an impact in cardiovascular protection and illustrate that this specific melon concentrate could be used to prevent or attenuate Western diet–related vascular dysfunction. More generally, the induction of endogenous defense implies that this melon concentrate could prevent alterations induced by different pathological situations in which oxidative stress is enhanced. Such a nutritional strategy is in line with the therapeutic guidelines and with the current concept that bioactive phytochemicals play a significant therapeutic role in attenuating oxidative damage induced by metabolic syndrome. However, the beneficial influence of this specific melon concentrate as a dietary supplementation has to be validated in humans.
